# Supplementary material for: AAV2-Retro–Mediated Gene Transfer Selectively Targets Outer Retinal Cells Following Intravitreal Injection
Source: Invest Ophthalmol Vis Sci. 2026 Mar 27;67(3):54. doi: 10.1167/iovs.67.3.54 (PMC13037736; doi:10.1167/iovs.67.3.54)
Supplement: Supplement 1 [file iovs-67-3-54_s001.pdf]

SUPPORTING INFORMATION

Title: AAV2-Retro-Mediated Gene Transfer Selectively Targets Outer Retinal Cells Following Intravitreal Injection

Authors: Chaimaa Kinane[1], Moxa Panchal[1], Pantelis Tsoulfas[4], Venu Talla[1], Kevin K. Park[1,2,3*]

Affiliations:

[1]Department of Ophthalmology, University of Texas Southwestern Medical Center

[2]Department of Neuroscience, University of Texas Southwestern Medical Center

[3]Peter O'Donnell Jr. Brain Institute, University of Texas Southwestern Medical Center

[4]Department of Neurosurgery, The Miami Project to Cure Paralysis, University of Miami Miller School of Medicine

*, corresponding author

Supplementary Figure

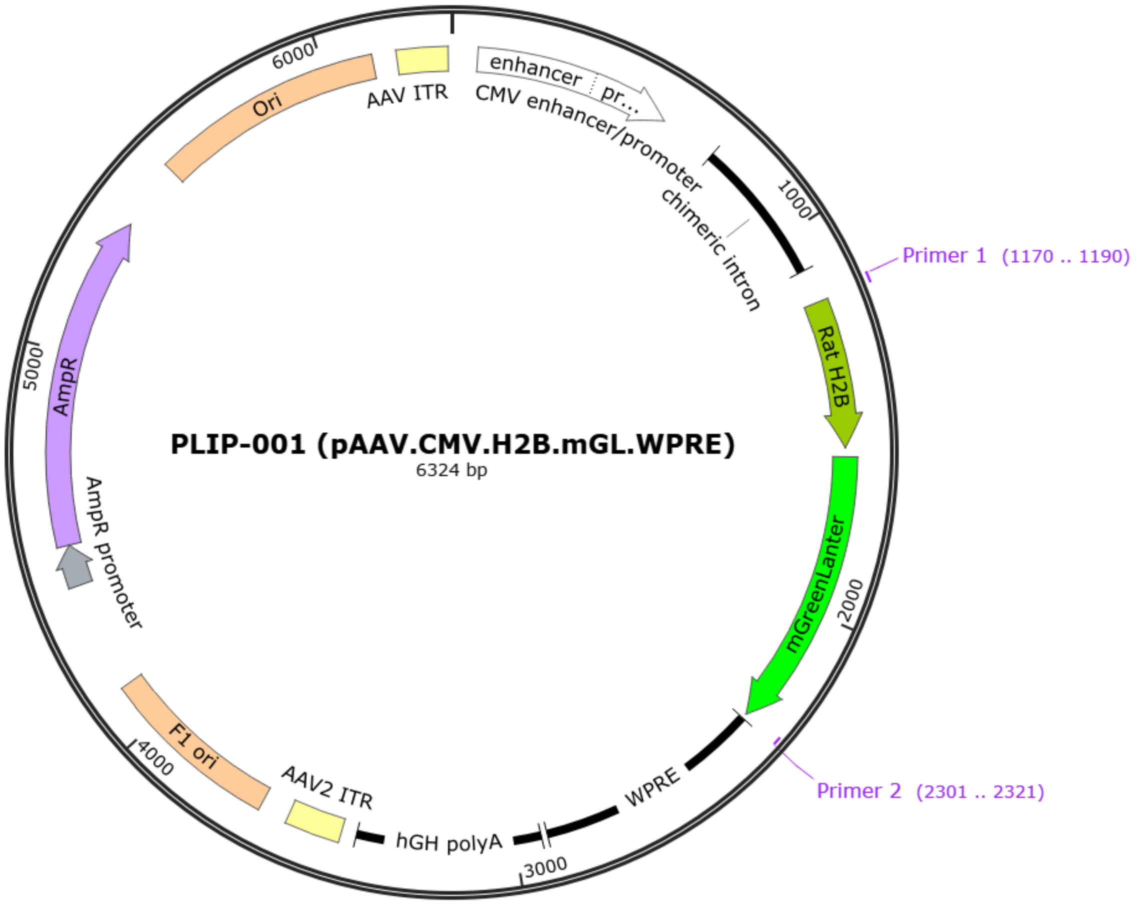

Figure S1: The Map of AAV2-retro-CMV-mGL vector. The transgene cassette comprises a CMV promoter driving mGL fused to H2B, followed by WPRE and hGH polyA, flanked by AAV2 ITRs.
